# Cerebral Glutamate Alterations Using Chemical Exchange Saturation Transfer Imaging in a Rat Model of Lipopolysaccharide-Induced Sepsis

**DOI:** 10.3390/metabo13050636

**Published:** 2023-05-08

**Authors:** Do-Wan Lee, Jae-Im Kwon, Hwon Heo, Chul-Woong Woo, Na Hee Yu, Kyung Won Kim, Dong-Cheol Woo

**Affiliations:** 1 Department of Radiology, University of Ulsan College of Medicine, Asan Medical Center, Seoul 05505, Republic of Korea; dwlee.mri@gmail.com (D.-W.L.); medimash@gmail.com (K.W.K.); 2 Department of Medical Science, Asan Medical Institute of Convergence Science and Technology, University of Ulsan College of Medicine, Asan Medical Center, Seoul 05505, Republic of Korea; kji1733@naver.com; 3 Nonclinical Research Center, QuBEST BIO Inc., Giheung-gu, Yongin-si 17015, Gyeonggi-do, Republic of Korea; ynh506@naver.com; 4 Department of Convergence Medicine, University of Ulsan College of Medicine, Asan Medical Center, Seoul 05505, Republic of Korea; heohwon@gmail.com; 5 Convergence Medicine Research Center, Asan Institute for Life Sciences, Asan Medical Center, Seoul 05505, Republic of Korea; wandj79@hanmail.net

**Keywords:** glutamate, chemical exchange saturation transfer, lipopolysaccharide, sepsis

## Abstract

Glutamate-weighted chemical exchange saturation transfer (GluCEST) is a useful imaging tool to detect glutamate signal alterations caused by neuroinflammation. This study aimed to visualize and quantitatively evaluate hippocampal glutamate alterations in a rat model of sepsis-induced brain injury using GluCEST and proton magnetic resonance spectroscopy (^1^H-MRS). Twenty-one Sprague Dawley rats were divided into three groups (sepsis-induced groups (SEP05, *n* = 7 and SEP10, *n* = 7) and controls (*n* = 7)). Sepsis was induced through a single intraperitoneal injection of lipopolysaccharide (LPS) at a dose of 5 mg/kg (SEP05) or 10 mg/kg (SEP10). GluCEST values and ^1^H-MRS concentrations in the hippocampal region were quantified using conventional magnetization transfer ratio asymmetry and a water scaling method, respectively. In addition, we examined immunohistochemical and immunofluorescence staining to observe the immune response and activity in the hippocampal region after LPS exposure. The GluCEST and ^1^H-MRS results showed that GluCEST values and glutamate concentrations were significantly higher in sepsis-induced rats than those in controls as the LPS dose increased. GluCEST imaging may be a helpful technique for defining biomarkers to estimate glutamate-related metabolism in sepsis-associated diseases.

## 1. Introduction

Sepsis is a complex systemic syndrome characterized by an imbalance between pro- and anti-inflammatory responses to invading pathogens [1] and is a major cause of morbidity and mortality in intensive care units worldwide [2]. Sepsis and its associated complications may progress to septic shock and multi-organ failure through uncontrolled production of proinflammatory cytokines [3]. Cerebral dysfunction is the initial symptom and frequent complication of sepsis, usually presenting as sepsis-associated encephalopathy (SAE), with an incidence rate of up to 70%, depending on the severity of the patient’s symptoms [2,4,5]. Moreover, SAE can affect brain cells and induce blood–brain barrier breakdown, intracellular metabolism dysfunction, brain cell death, and brain injuries [6]. Therefore, biophysical assessment of SAE in cerebral metabolism through observational and follow-up studies might be used as an important diagnostic tool and an effective treatment strategy to prevent brain damage [5].

Various studies using medical imaging techniques, such as magnetic resonance spectroscopy (MRS), magnetic resonance imaging (MRI), single-photon emission tomography, and positron emission tomography (PET), have been conducted [7,8,9,10,11]. These imaging techniques can detect the distribution of glutamate (Glu), an in vivo brain neurotransmitter, and changes in Glu levels [7,8,9,10,11]. Conventional magnetic resonance (MR)-based imaging techniques present morphological information but are of limited value for evaluating more specific and reproducible information regarding cellular changes and biochemical abnormalities [12]. High-resolution in vivo proton MRS (^1^H MRS) (≥7 T) is a unique method that can detect changes in neurochemical compounds involved in brain diseases and is useful in identifying significant disease biomarkers [11,13]. Although MRS has sufficient sensitivity to detect neurotransmitters in many areas of the brain, it lacks adequate spatial resolution, which is necessary for quantitative brain mapping and lesion visualization [14].

Chemical exchange saturation transfer (CEST) MRI has been introduced as a new contrast enhancement technique that provides molecular information for diagnosing pathological tissues and detecting molecular responses to treatment [15,16]. In addition, CEST imaging can indirectly detect metabolic signals (including Glu, amide proton, creatine, myo-inositol, and glycogen) with exchangeable endogenous protons and exchange-related properties [17]. Among endogenous agents, Glu CEST (GluCEST), which is sensitive to changes in Glu concentration resonating at 3.0 ppm from water (0 ppm), has been widely used in cerebral diseases, such as epilepsy, ischemia, tumors, and psychiatric disorders [14,18,19,20,21,22].

We aimed to evaluate in vivo Glu signal changes within the hippocampal region in a rat model of sepsis-induced brain injury using well-validated in vivo ^1^H MRS and GluCEST imaging at 7 T. To our knowledge, this study is the first to assess GluCEST imaging as a potential marker of the response to sepsis. In addition, we examined immunohistochemical and immunofluorescence staining to observe the immune response and activity in the hippocampal region after lipopolysaccharide (LPS) exposure.

## 2. Materials and Methods

### 2.1. Animal Models

All experiments were conducted to minimize the number of animals used in the study and the pain and suffering of all experimental animals. The study complied with the principles outlined in the Animal Research: Reporting of In Vivo Experiments guidelines and Basel Declaration, including the replacement, reduction, and refinement (3R) concept. Moreover, all animal care and experiments were conducted with the approval of the Animal Care and Use Committee of the Asan Medical Center of the University of Ulsan Medical School (approval date: 25 February 2020; permit code: 2020-13-056).

Starting at 8 weeks of age, 21 Sprague Dawley rats were obtained from Orient Bio, Inc. (Seongnam, Kyunggi-do, Korea) and divided into three groups (sepsis-induced groups (SEP05, *n* = 7 and SEP10, *n* = 7) and control (CTRL, *n* = 7) group). The animals were pair-housed in standard plastic cages and maintained on a 12 h light–dark cycle at an ambient temperature of 23–24 °C. Before the start of the experiments, the animals were allowed free access to food and water for 1 week.

Sepsis was induced through a single intraperitoneal injection of LPS (from *Escherichia coli* O26:B6; L8274; Sigma-Aldrich, St. Louis, MO, USA) at doses of 5 mg/kg (SEP05) and 10 mg/kg (SEP10) [23,24]. An equal volume of vehicle (sterile endotoxin-free PBS) as that in SEP05 and SEP10 groups was administered intraperitoneally to the CTRL rats. All animals underwent MRI 24 h after injection of a single dose of LPS or vehicle.

### 2.2. MRI Scan

MRI experiments were conducted using a 7 T horizontal-bore PharmaScan 70/16 scanner (Bruker BioSpin GmbH, Ettlingen, Germany) with a 400 mT/m self-shielding gradient system and an actively decoupled cross-coil setup, which consisted of a 72 mm body coil for excitation and 25 mm single-loop rat brain surface coil. During the MRI scans, the animals were anesthetized using medical air (1.0 L/min) with isoflurane (1.5–3.0% for induction and 2.0% for maintenance) in a mixture of 70% N_2_O and 30% O_2_ administered through a nose cone and were immobilized with ear-bars during scanning. Body temperature was maintained at approximately 37 °C using a warm-water circulation system in a flat bed positioned around the rat’s body. Physiological parameters, including heart and respiratory rates, were monitored using an animal respiratory gating system (SA Instruments, Stony Brook, NY, USA).

Before MRI acquisition, the B_0_ field was corrected through localized high-order shimming using a region of interest (ROI, 34 × 34 × 34 mm^3^) covering the whole cerebral area to achieve a homogeneous B_0_ field and a high signal-to-noise ratio in the MRI system. Subsequently, the radio frequency (RF) field and center frequency were calibrated using the prescan protocol. GluCEST and ^1^H MRS scans were conducted sequentially for each rat.

In vivo GluCEST data were collected from a single slice in which the hippocampal region was well observed, and CEST parameters were applied as follows: fat-suppressed, turbo-rapid acquisition with relaxation enhancement sequence with a continuous-wave RF saturation pulse, repetition time (TR) = 4.2 s, echo time (TE) = 36.4 ms, RF saturation power = 3.6 μT, RF saturation time = 1 s, 96 × 96 matrix size, 30 × 30 mm^2^ field-of-view (FOV), and 1.5 mm slice thickness [14,18,25]. Z-spectra with 25 frequency offsets from –6 to +6 ppm (intervals of 0.5 ppm) and an unsaturated reference image (S_0_) were obtained from the selected single-slice image. To correct the B_0_ field inhomogeneity, water saturation shift referencing (WASSR) Z-spectra with 33 frequency offsets from –0.8 to +0.8 ppm (intervals of 0.05 ppm) using 0.3 μT RF saturation power were applied [26]. The B_1_ map was generated from two images obtained using the dual flip-angle (30° and 60°) method [18].

For the single voxel placement of ^1^H MRS, anatomical T_2_-weighted MR images with TR/TE = 5250/66 ms, FOV 25 × 25 mm^2^, 256 × 256 matrix size, 11 ms echo spacing, and 2 averages were obtained in all three orthogonal planes. A spin-echo-based, point-resolved spectroscopy pulse sequence with water-selective suppression was used for the hippocampal region. All parameters were as follows: variable power and optimized relaxation delays method, volume of interest = 2 × 2 × 3 mm^3^ (12.0 µL), TR/TE = 5000/16.3 ms, 5000 Hz spectral width, 256 average, 2048 data points, and total scan time = 23 min 15 s.

### 2.3. Data Analysis

All CEST data processing and analysis were performed using custom-written scripts in MATLAB 2022b (MathWorks, Natick, MA, USA). To correct the B_0_ inhomogeneity, the Z-spectra from the WASSR data for each voxel were fitted, and the water signal in the chemical shift was reset to 0 ppm to obtain the frequency shift information in each voxel. The relative B_1_ values were calculated using the B_1_ map. The GluCEST signal was calculated by subtracting the normalized magnetization signal at 3.0 ppm from the magnetization at the corresponding reference frequency symmetrically up-field from water; GluCEST (%) = 100 × (S_sat@−3.0 ppm_ − S_sat@+3.0 ppm_) ÷ S_sat@−3.0 ppm_ [18], where S_sat@−3.0 ppm_ and S_sat@+3.0 ppm_ are the water signals with a saturation pulse at offsets ±3 ppm from the water resonance. GluCEST signals in all rats were carefully quantified based on a manually drawn ROI in the hippocampal region.

In vivo ^1^H MRS raw data were quantified using a linear combination of models (v. 6.3-1D; copyright: Stephen W. Provencher, Stephen Provencher Inc., Oakville, Canada) in a fully automated pipeline and a set of simulated basis sets, including 18 metabolites as follows: alanine, aspartate, creatine (Cr), gamma-aminobutyric acid, glucose, Glu, glutamine (Gln), glutathione, glycerophosphocholine (GPC), glycine, lactate, N-acetylaspartate (NAA), N-acetylaspartylglutamate (NAAG), myoinositol, phosphocholine (PCh), phosphocreatine (PCr), scylloinositol, taurine, total NAA = NAA + NAAG, glutamine and Glu complex (Glx) = Glu + Gln, total Cr = Cr + PCr, and total choline (Cho) = GPC + PCh. The ^1^H MRS signal from the hippocampal region was calculated with water scaling for quantification of metabolic concentrations (µmol/g) and eddy current compensation. All metabolite peaks were fitted in the chemical shift from 4.0 to 0.3 ppm. 

### 2.4. Hematoxylin and Eosin Staining

After MRI scans, all animals were anesthetized and fixed through cardiac perfusion using 4.0% paraformaldehyde (PFA) (Biosesang, Seongnam, Republic of Korea). The brain tissues were obtained and then soaked in 4% PFA for 24 h. Paraffin-embedded tissues were sectioned into 3 μm thickness and mounted on slides coated with poly-L-lysine. Finally, the slides were stained with hematoxylin (for 10 min) and eosin (for 5 min) (H&E staining) at room temperature. The pathological features of the hippocampal tissues were observed using an automated quantitative pathology imaging system (Leica, Wetzlar, Germany). H&E staining was performed as previously described [27,28].

### 2.5. Immunofluorescence (NeuN, DAPI, and Iba-1)

Immunofluorescence staining was performed as previously described [27,29,30]. The sectioned tissues were deparaffinized in a dry oven at 60 °C and then rehydrated. The tissues were immersed to prevent nonspecific binding with normal goat serum (Jackson ImmunoResearch, West Grove, PA, USA) for 1 h at room temperature and incubated with primary antibodies using anti-Iba1 (1:100; Abcam, Cambridge, UK) and antineuronal nuclear (1:100; Millipore, Burlington, MA, USA) overnight at 4 °C. The following day, the incubated tissues were rinsed thrice using 1× phosphate-buffered saline with 0.01% Tween 20 (PBST) (Sigma-Aldrich, St. Louis, MO, USA) for 5 min, and then fluorescent secondary antibodies were treated with AlexaFluor-488 goat antirabbit (1:500; Invitrogen, Waltham, MA, USA) and AlexaFluor-594 goat antimouse (1:500; Invitrogen, Waltham, MA, USA) for 1 h at room temperature. After incubation, the tissues were rinsed thrice with 1× PBST for 5 min and dehydrated. Finally, the tissues were cover-slipped using a mounting medium containing 4′,6-diamidino-2-phenylindole (DAPI; Abcam, Cambridge, UK). Images were acquired using Vectra 3 (Akoya Bioscience, Marlborough, MA, USA) under a microscope at a magnification of 200×.

### 2.6. Statistical Analysis

All statistical analyses were performed using PASW Statistics 18 (SPSS Inc., IBM Company, Chicago, IL, USA). The Kolmogorov–Smirnov and Shapiro–Wilk tests were used to test the normality of the GluCEST and ^1^H MRS data in the three groups [31]. The *p* values for GluCEST and ^1^H MRS data normality in all groups were above 0.05 (*p* < 0.169 for the Kolmogorov–Smirnov test and *p* < 0.594 for the Shapiro–Wilk test), indicating that the data were normally distributed. The results of the present study described one independent variable (dose of LPS) and two dependent variables (GluCEST values and Glu concentrations in the hippocampal region), so one-way analysis of variance was used [32]. As the sample sizes for each group were equal, the Tukey’s post hoc test was performed for pairwise comparisons between the group means [33]. Statistical differences were assumed to be significant for *p* values < 0.05.

G*Power 3.1.9.7 software was used to calculate the animal sample size required for the present study. The sample size suitable for the experiment was computed by applying α  =  0.05, power (1 − β) = 0.8, and effect size = 0.8. In the G*Power analysis, an actual power of 0.86285 was used. A required sample size of seven animals per group (total sample size = 21) was obtained.

## 3. Results

**Figure 1:**
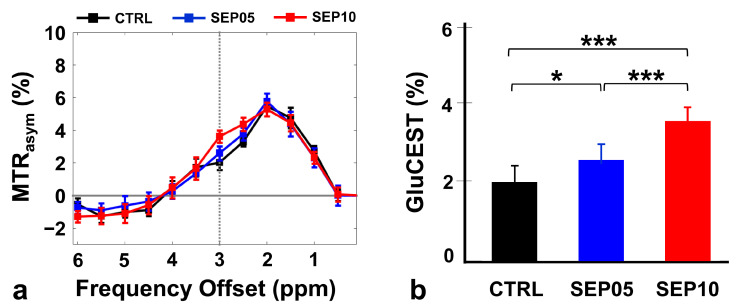
Magnetization transfer ratio asymmetry (MTR_asym_) spectra (**a**) in the hippocampal region in the control (CTRL; *n* = 7) and sepsis-induced groups with doses of 5 mg/kg (SEP05; *n* = 7) and 10 mg/kg (SEP10; *n* = 7). The dotted line indicates the point at 3.0 ppm used for the quantification of glutamate-weighted chemical exchange saturation transfer (GluCEST). The quantified GluCEST signals (%) presented as bar graphs represent the mean + standard error of the mean for each group (**b**). * *p* < 0.05 and *** *p* < 0.001.

Figure 1 shows the magnetization transfer ratio asymmetry (MTR_asym_) curves (a) and quantified GluCEST signals at 3.0 ppm (b) in the rat hippocampus in each group (CTRL, *n* = 7; SEP05, *n* = 7; and SEP10, *n* = 7). The MTR_asym_ curves showed distinct differences among the groups. As the LPS dose increased, the MTR_asym_ values were higher than those in the CTRL group. Moreover, as the frequency offset increased (approximately ≥ 4.0 ppm), the MTR_asym_ data in all groups were negative, which might be the effect of upfield nuclear Overhauser enhancement [34,35]. The quantified GluCEST signals differed significantly among the three groups. The mean GluCEST values and standard deviation (SD) are shown, as follows (Figure 1b): CTRL (2.03 ± 0.45%) vs. SEP05 (2.60 ± 0.43%) (*p* < 0.05), SEP05 vs. SEP10 (3.62 ± 0.38%) (*p* < 0.001), and CTRL vs. SEP10 (*p* < 0.001).

**Figure 2:**
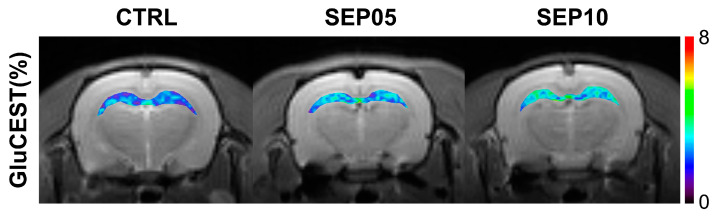
Reconstructed GluCEST maps overlaid on unsaturated images (S_0_) targeting the hippocampal region of the representative rats in the CTRL and sepsis-induced groups with doses of 5 mg/kg (SEP05) and 10 mg/kg (SEP10).

Figure 2 illustrates the bilateral cerebral mapping results of GluCEST signals in representative rats, focusing on the hippocampal region overlaid on the unsaturated CEST image (S_0_) in each group. Compared to the CTRL group, the GluCEST signals in the hippocampal region showed changes in hyperintensity with increasing doses of LPS.

**Figure 3:**
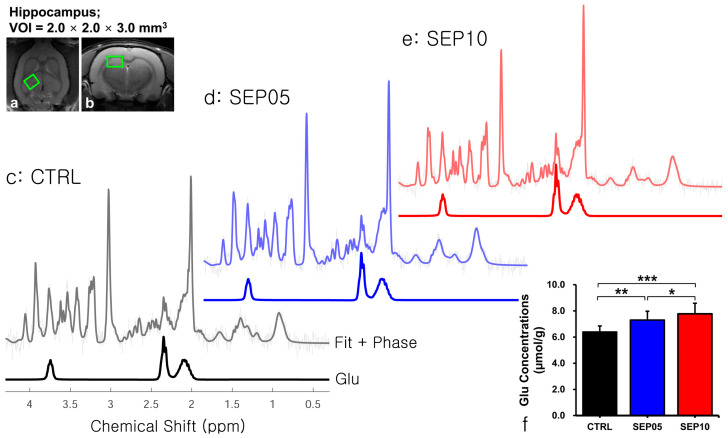
LCModel-fitted results of the proton magnetic resonance spectra in the hippocampal region (**a**,**b**) acquired from representative rats from the control group (CTRL) (**c**) and sepsis-induced groups with LPS doses of 5 mg/kg (SEP05) (**d**) and 10 mg/kg (SEP10) (**e**). The bar chart (**f**) shows the mean glutamate (Glu) concentration in the rat hippocampus in each group (CTRL, *n* = 7; SEP05, *n* = 7; and SEP10, *n* = 7), and the vertical lines on each of the bars represent the standard deviation (ppm, part per million; VOI, volume of interest; significance levels: * *p* < 0.05, ** *p* < 0.01, and *** *p* < 0.001).

Figure 3 shows the voxel placement in the hippocampal region (Figure 3a,b) and presents the spectral fitting results of ^1^H-MRS data in a representative rat from each group (Figure 3c–e). Visual inspection of the proton spectra indicated that the Glu peak area was slightly different among the three groups and showed higher intensities with an increasing dose of LPS than that in the controls. The quantified Glu concentrations differed significantly among the three groups. The mean Glu concentrations and SD are shown as follows (Figure 3f): CTRL (*n* = 7; 6.401 ± 0.450 µmol/g) vs. SEP05 (*n* = 7; 7.303 ± 0.671 µmol/g) (*p* < 0.01), SEP05 vs. SEP10 (*n* = 7; 7.773 ± 0.812 µmol/g) (*p* < 0.05), and CTRL vs. SEP10 (*p* < 0.001). Overall, the ^1^H-MRS data demonstrated a similar trend to the GluCEST data, showing high Glu concentrations as the LPS administration dose increased.

**Figure 4:**
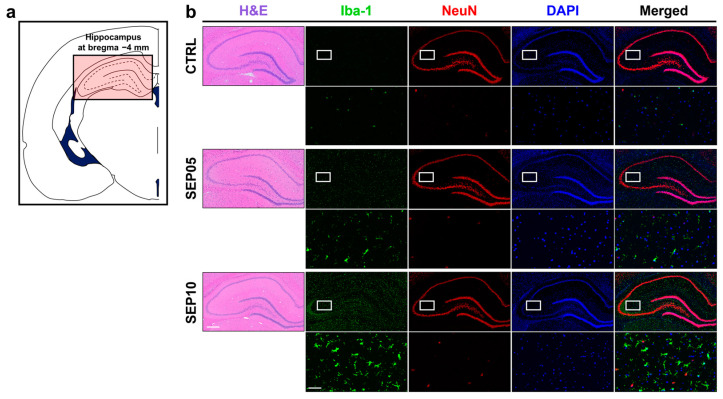
(**a**) Schematic representation of a coronal section of a rat brain highlighting the middle segmentation of the hippocampal region under examination. (**b**) Sections from the hippocampus are stained with hematoxylin and eosin (H&E) (left column), Iba-1 (green), neuronal nuclear (NeuN) (red), and 4′,6-diamidino-2-phenylindole (DAPI) (blue). The images in the second row of each group represent the corresponding regions of interest (white boxes) displayed at a higher magnification (scale bar = 25 µm). The sections are sourced from representative rats in the control (CTRL) and sepsis-induced groups (LPS doses of 5 mg/kg (SEP05) and 10 mg/kg (SEP10)). Scale bar in H&E: 200 µm.

Figure 4 shows the histological approach of the hippocampal region in a representative rat from each group. Representative H&E staining and immunofluorescence show the biological characteristics of sepsis-induced cerebral metabolism in the hippocampal region (Figure 4b).

## 4. Discussion

Although the pathological process of SAE remains unclear, early diagnosis and treatment are important to reduce mortality and disability due to cerebral injury in patients with sepsis. The present study showed that GluCEST imaging and ^1^H MRS techniques have a high sensitivity to changes in Glu signals in vivo in the hippocampal region of rats with SAE. Glutamate distribution in the rat hippocampal region was also determined. In addition, we tested cerebral neuroinflammation status and biochemical changes during the SAE using immunohistochemical and immunofluorescence staining.

Since its introduction, CEST has been used in numerous clinical applications for various diseases, such as epilepsy, ischemia, tumors, and psychiatric disorders, to detect in vivo signal changes of specific endogenous agents (for example, proteins and neurotransmitters, such as Glu) [14,18,19,20,21,22]. However, to date, only a small number of in vivo studies have assessed signal changes in cerebral metabolites using MR-based imaging techniques in patients with SAE or animal models. To our knowledge, no previous studies have evaluated changes in the GluCEST signal caused by SAE. The present study clearly showed in vivo Glu changes in sepsis-induced lesions, demonstrating higher GluCEST values and Glu concentrations (^1^H MRS) than those in the CTRL group as the LPS dose increased. Regarding the GluCEST signals quantified in this study, we attempted to minimize the factors that influence GluCEST signal formation. As several previous studies have indicated [18,25,26], B_0_ and B_1_ field inhomogeneities may affect signal drift by overall Z-spectrum shift, resulting in inaccurate signal quantification. To address these factors, we applied the WASSR method and relative B_1_ map calculation, respectively, to correct for field inhomogeneity in the whole frequency offset of the GluCEST data. We used a relatively high B_1_ saturation power (3.6 μT in the present study) and short saturation time to observe the Glu-weighted signals in the 7 T system. In addition, to minimize the change in pH, which would affect GluCEST signaling, the body temperature of the experimental animals was maintained (37 °C) in this study. Therefore, based on the factors mentioned above, there were significant differences in GluCEST values among the three groups, which can be solely attributed to the differences in Glu concentrations.

Several studies using ^1^H MRS have reported quantitative evaluations of cerebral metabolite changes in SAE [36,37,38]. Wen et al. [38] demonstrated a significant decrease in the NAA/Cr ratio in sepsis-induced rats based on the LPS administration model compared to controls, although there was no difference in T2-weighted MR images. Several sepsis-induced animal studies using the cecal ligation and perforation surgical model showed significant differences in the values of various metabolite ratios compared to the baseline or controls, such as increased Glx/Cr [37] and mI/Cr [37] ratios and decreased NAA/Cr [37] and NAA/Cho [36] ratios. In previous conventional ^1^H MRS studies, cerebral metabolite changes in various diseases were quantified using the (specific metabolites)/Cr ratio method [39,40]. However, in addition to the relative quantification of these metabolites, the evaluation of absolute concentration based on internal standards (water scaling) might be helpful in interpreting the biological characteristics of the disease [41]. Contrary to the results of previous studies, the present study only quantified changes in Glu concentration using internal standards and observed distinct differences among the three groups. Kitagawa et al. [42] also demonstrated increased Glu levels in LPS-treated rats compared to controls and validated the increased microglial activation using immunofluorescent staining using an Iba-1 antibody. As is well known, the morphology of microglia can be visualized through the staining of Iba-1, which is a specific marker for pan-microglia [43]. Microglia are macrophages present in the central nervous system that orchestrate the inflammatory response in the brain [44]. Therefore, neuroinflammation is necessary to maintain immune homeostasis by inducing repair mechanisms [45]. However, failure to maintain homeostasis might be interpreted as a potentially harmful mechanism of brain injury [45,46]. Recent studies have demonstrated that increased Glu concentrations are often observed in neuronal inflammatory diseases and may be generated by an activated immune system and microglial reactivity during neuroinflammation [47,48,49]. In addition, Moraes et al. [46] suggested that activated microglia constitute a hallmark of SAE and may be involved in associated synaptic deficits.

However, this study presented some limitations. First, to extend the anatomical coverage of GluCEST imaging for detecting signals in SAE regions, multi-slice or three-dimensional GluCEST imaging is required. Further studies dealing with multiple regions of the brain susceptible to SAE, including gray and white matter regions, may provide information on the role of in vivo Glu in sepsis-related diseases and corroborate the results of this study. Second, because we focused on detecting changes in GluCEST values and Glu concentrations in a rat model of sepsis-induced brain injury, neurochemical profiles other than those of Glu were not evaluated. As previously reported [36,37,38], further studies are required for the quantitative evaluation of various neurochemical profiles, such as Cr, NAA, Glx, and Cho, in order to identify new biomarkers. Third, several previous studies have identified key cerebral markers in SAE using dynamic 18F-fluorodeoxyglucose PET with computed tomography, multi-parametric MR imaging (including diffusion-weighted or tensor imaging), and resting-state functional MRI [5,37]. Therefore, further studies are needed to evaluate the origin of Glu signals, as well as the dynamic metabolic activity, and to determine the severity of SAE at various time points, including during the early stages of sepsis progression. Fourth, since we did not quantitatively analyze the signal intensity in the immunofluorescence staining data, statistical significance could not be determined. Quantitative evaluation of neuroinflammation markers in the blood, as well as in various histological stainings of various brain regions, should be conducted in future studies to corroborate these results. Finally, the number of experimental animals used in this study was relatively small, although the present results showed that the changes in Glu signals were significant among the three groups. Further studies with a larger number of animal samples modeling SAE are required to generate more reliable results.

## 5. Conclusions

Glutamate-weighted 7 T MRI was adequately sensitive in detecting in vivo changes in the hippocampal region in sepsis-induced cerebral injuries. Significant contrast changes in Glu-weighted metrics coupled with the histologic characteristics of sepsis-induced cerebral injuries indicate the potential usefulness of GluCEST imaging in observing neuroinflammatory processes.

## Data Availability

The data that support the findings of this study are available from the corresponding author upon reasonable request. Data is not publicly available due to privacy or ethical restrictions.
